# Supplementary material for: Impact of a short online course on the accuracy of non-ophthalmic diabetic retinopathy graders in recognising glaucomatous optic nerves in Vietnam
Source: BMJ Open. 2023 Nov 9;13(11):e076623. doi: 10.1136/bmjopen-2023-076623 (PMC10649381; doi:10.1136/bmjopen-2023-076623)
Supplement: Author's manuscript [file bmjopen-2023-076623.draft_revisions.pdf]

BMJ Open

BMJ Open is committed to open peer review. As part of this commitment we make the peer review history of every article we publish publicly available.

When an article is published we post the peer reviewers' comments and the authors' responses online. We also post the versions of the paper that were used during peer review. These are the versions that the peer review comments apply to.

The versions of the paper that follow are the versions that were submitted during the peer review process. They are not the versions of record or the final published versions. They should not be cited or distributed as the published version of this manuscript.

BMJ Open is an open access journal and the full, final, typeset and author-corrected version of record of the manuscript is available on our site with no access controls, subscription charges or pay-per-view fees (http://bmjopen.bmj.com).

If you have any questions on BMJ Open's open peer review process please email info.bmjopen@bmj.com

BMJ Open

A short online course successfully trains non-ophthalmic diabetic retinopathy graders to recognize glaucomatous optic nerves in Vietnam

Journal:	*BMJ Open*
Manuscript ID	bmjopen-2023-076623
Article Type:	Original research
Date Submitted by the Author:	12-Jun-2023
Complete List of Authors:	Olawoye, Olusola; Queens University Belfast, Dentistry and Biomedical Sciences; University of Ibadan College of Medicine, Ophthalmology<br>Ha, Thu Huong; ORBIS International<br>Pham, Ngoc; ORBIS International<br>Nguyen, Lam; Hanoi Medical University<br>Cherwek, David; ORBIS International, Global Programs<br>Fowobaje, Kayode Raphael; University of Ibadan, Department of Epidemiology and Medical Statistics<br>Ross, Craig; Centre for Eye Research Australia<br>Coote, Michael; Centre for Eye Research Australia<br>Chan, Ving Fai; Queens University Belfast, Dentistry and Biomedical Sciences<br>Kahook, Malik; University of Colorado at Colorado Springs<br>Peto, Tunde; Queen's University Belfast Faculty of Medicine Health and Life Sciences<br>Azuara-Blanco, Augusto ; Queens University Belfast , Centre for Public Health<br>Congdon, Nathan; Queen's University Belfast, Ophthalmology and Public Health
Keywords:	Glaucoma < OPHTHALMOLOGY, EPIDEMIOLOGY, PUBLIC HEALTH

SCHOLARONE™
Manuscripts

I, the Submitting Author has the right to grant and does grant on behalf of all authors of the Work (as defined in the below author licence), an exclusive licence and/or a non-exclusive licence for contributions from authors who are: i) UK Crown employees; ii) where BMJ has agreed a CC-BY licence shall apply, and/or iii) in accordance with the terms applicable for US Federal Government officers or employees acting as part of their official duties; on a worldwide, perpetual, irrevocable, royalty-free basis to BMJ Publishing Group Ltd ("BMJ") its licensees and where the relevant Journal is co-owned by BMJ to the co-owners of the Journal, to publish the Work in this journal and any other BMJ products and to exploit all rights, as set out in our licence.

The Submitting Author accepts and understands that any supply made under these terms is made by BMJ to the Submitting Author unless you are acting as an employee on behalf of your employer or a postgraduate student of an affiliated institution which is paying any applicable article publishing charge ("APC") for Open Access articles. Where the Submitting Author wishes to make the Work available on an Open Access basis (and intends to pay the relevant APC), the terms of reuse of such Open Access shall be governed by a Creative Commons licence – details of these licences and which Creative Commons licence will apply to this Work are set out in our licence referred to above.

Other than as permitted in any relevant BMJ Author's Self Archiving Policies, I confirm this Work has not been accepted for publication elsewhere, is not being considered for publication elsewhere and does not duplicate material already published. I confirm all authors consent to publication of this Work and authorise the granting of this licence.

For peer review only

Title: A short online course successfully trains non-ophthalmic diabetic retinopathy graders to recognize glaucomatous optic nerves in Vietnam

Authors: Olusola Olawoye,[1,2] Thu Huong Ha,[3] Ngoc Pham,[3] Lam Nguyen,[4] David Hunter Cherwek,[5] Kayode Raphael Fowobaje,[6] Craig Ross, [7] Michael Coote,[7] Ving Fai Chan,[1] Malik Y. Kahook,[8] Tunde Peto,[1] Augusto Azuara Blanco,[1] Nathan Congdon.[1,5,9]

Affiliations:

[1]Centre for Public Health, School of Medicine, Dentistry and Biomedical Sciences. Queens University Belfast, United Kingdom

[2]College of Medicine University of Ibadan Nigeria

[3]Orbis International, Vietnam

[4]Hanoi Medical University, Vietnam

[5] Orbis International, NY, United States

[6]Department of Epidemiology and Medical Statistics, Faculty of Public Health, College of Medicine, University of Ibadan, Nigeria

[7]Centre for Eye Research Australia, East Melbourne, Australia

[8]Sue Anschutz-Rodgers Eye Centre, University of Colorado School of Medicine

[9]Zhongshan Ophthalmic Centre, Sun Yat-sen University, Guangzhou, China

Funding support: eXcellence in Ophthalmology and Vision Awards (XOVA Novartis)

Running Title: Online training for non-ophthalmologists to recognize glaucoma

Conflict of Interest: Congdon serves as a paid consultant for Belkin Vision, a company selling devices for the treatment of glaucoma. Kahook has an equity interest in SpyGlass Pharma and receives royalties from New World Medical and Alcon. Olusola Olawoye is a Consortium for Advanced Research and Training in Africa fellow.

Corresponding Author: Olusola Olawoye

Address: Centre for Public Health, School of Medicine, Dentistry and Biomedical Sciences. Queen's University Belfast, United Kingdom E-mail: solaolawoye@yahoo.com

Keywords: Glaucoma, screening, online course, diabetic retinopathy grading, non-ophthalmic graders, training

For peer review only - http://bmjopen.bmj.com/site/about/guidelines.xhtml

Abstract

Purpose: To test an online training course for non-ophthalmic diabetic retinopathy (DR) graders for recognition of glaucomatous optic nerves in Vietnam.

Methods: This was an uncontrolled, experimental, before-and-after study in which 43 non-ophthalmic DR graders underwent baseline testing on a standard image set, completed a self-paced, online training course and were retested using the same photographs presented randomly. 29 local ophthalmologists completed the same test without the training course. DR graders then underwent additional one-one training by a glaucoma specialist and were re-tested. Test performance (% correct, compared to consensus grades from 4 fellowship-trained glaucoma experts), sensitivity, specificity, positive and negative predictive value, and area under the receiver operating (AUC) curve, were computed.

Results: Mean age of DR graders (32.6±5.5 years) did not differ from ophthalmologists (32.3±7.3 years, p=0.13). Online training required a mean of 297.9 (SD 144.6) minutes. Graders' mean baseline score (33.3% ± 14.3%) improved significantly after training (55.8% ±12.6%, P <0.001), and post-training score did not differ from ophthalmologists (58.7±15.4%, P=0.384]. Grader sensitivity did not differ before [85.5% (95% CI 83.5 – 87.3)] vs after [80.4% (78.3 – 82.4)] training, while specificity improved significantly [47.8 (44.9 – 50.7) vs 79.8 (77.3 – 82.0), p<0.001]. Grader AUC also improved after training [66.6 (64.9 – 68.3) to 80.1 (78.5 – 81.6), p<0.001]. Additional one-one grader training by a glaucoma specialist did not further improve grader scores.

Conclusion: Non-ophthalmic DR graders can be trained to recognise glaucoma using a short online course in this setting, with no additional benefit from more expensive one-one training.

For peer review only - http://bmjopen.bmj.com/site/about/guidelines.xhtml

Strengths and Limitations of this study

Strengths

- Strengths of this study include an approach to assessing the impact of training which included both a before-after comparison and external validation against the performance of local ophthalmologists.
- Both the course and the assessment test reflected input from clinical experts, specialists in image-based screening and those engaged professionally in capacity-building for the recognition of glaucomatous optic nerves.
- A number of widely-used indices of screening accuracy were computed, allowing us to elucidate specific benefits of training in improving specificity.

Limitations

- The generalizability of our results to other cadres of trainees and other settings is uncertain.
- Further research is needed to better understand the practicality of this training model within the context of a full glaucoma screening program.

For peer review only

For peer review only - http://bmjopen.bmj.com/site/about/guidelines.xhtml

Synopsis:

After 5-hour online training in recognizing glaucomatous discs, scores of non-ophthalmic diabetic retinopathy graders doubled, and did not differ from local ophthalmologists. Intensive one-to-one training did not further improve performance.

For peer review only

For peer review only - http://bmjopen.bmj.com/site/about/guidelines.xhtml

Key Message

What is already known on this topic: Little is known about the efficacy of interventions to train existing non-ophthalmic diabetic retinopathy (DR) graders to deliver accurate glaucoma screening on available retinal images.

What this study adds: Our study found that after a 5-hour, self-paced online training course in recognizing glaucomatous optic nerves, scores of non-ophthalmic DR graders doubled, and did not differ from local ophthalmologists. More resource-intensive one-to-one training by specialists did not further improve performance.

How this study might affect research, practice or policy: This training model may leverage existing DR screening capacity to improve glaucoma detection in low resource settings

For peer review only

For peer review only - http://bmjopen.bmj.com/site/about/guidelines.xhtml

Introduction

Glaucoma is the leading cause of irreversible blindness globally.[1,2] The number of persons living with glaucoma has increased from 64.3 million in 2013 to 76.0 million in 2020,[3] and is projected to rise a further 74% by 2040 due to an ageing and growing global population. Asia accounts for 60% of the world's total glaucoma cases, followed by Africa at 13%.[3] The number of persons blind due to glaucoma increased by 0.8 million between 1990 and 2010, with most of this rise occurring in low and middle-income countries (LMICs).[4] This trend is likely to continue without new and more effective screening strategies appropriate for low-resource settings.

Glaucoma often progresses unnoticed until central visual acuity is permanently compromised, therefore early detection of the disease, before symptoms develop is key in reducing the impact of visual loss in these patients. The rate of undiagnosed glaucoma is high, especially in LMICs, where approximately 95% of those affected are not aware of having the disease.[5,6]

Screening of any disease is a form of secondary prevention, with the goal of preventing undesirable outcomes. In the context of glaucoma, screening is designed to promote diagnosis, and treatment if necessary, during the asymptomatic stage in order to prevent further vision loss and blindness. So far, glaucoma screening has not been found to be cost effective in high income countries.[7,8]

Recently, however, a decision-analytic Markov model has been used to show that population screening for both primary open angle glaucoma (POAG) and primary angle closure glaucoma (PACG) was cost effective in both rural and urban China.[9] John et al [10] reported that community screening for glaucoma in India would prevent 2,190 person years of blindness over a 10-year period. They concluded that community population screening might be cost effective if targeted at persons aged 40-69-years, and if implemented in urban areas.

For peer review only - http://bmjopen.bmj.com/site/about/guidelines.xhtml

Unlike screening programs for glaucoma, diabetic retinopathy (DR) screening is well established in many high-income countries, and increasingly in LMICs as well. It would seem logical that screening for two major blinding eye diseases, glaucoma and DR, using the same equipment and personnel would be more cost effective. It may be possible to train non-ophthalmic graders who are currently screening for DR to recognize glaucoma using the colour fundus images, reducing the diagnostic burden on scarce, highly trained health care providers in low-resource settings.

The aim of the current study is to develop and test an online course to train nurses, technicians, and non-ophthalmologist physicians working as DR graders in a non-governmental organization (NGO) program in Vietnam to recognize glaucomatous optic nerves from ophthalmic images. The pre- and post-training performance of graders are assessed on a standard test set of optic nerve images drawn from a variety of sources, and compared with the performance of local ophthalmologists not undergoing the same training.

Methods

Approval for the study was obtained from the Ethical Review Board of the Queen's University Belfast in the United Kingdom, and the Ethics Committee of the Hanoi Medical College in Vietnam before the commencement of the study. Participating DR graders and ophthalmologists in Vietnam all provided written informed consent before recruitment into the study.

This is an uncontrolled, experimental, before-and-after study in which 43 non-ophthalmic DR graders were trained to screen for glaucoma using optic nerve photos. The online training course was developed specifically for the study and focused on a standardized approach to the pattern recognition of glaucomatous damage to the optic nerve. The study flow was as follows:

For peer review only - http://bmjopen.bmj.com/site/about/guidelines.xhtml

- Non-ophthalmic graders in an NGO DR program (DR graders) were consented, enrolled and underwent testing on the standard image set at baseline.
- The DR graders then completed the self-paced, online training course. Participants were encouraged to attempt practice questions placed at the end of each module of the course, and those failing to answer these correctly were asked to review the course again.
- DR graders then repeated the test on the standard image set. Images were identical to those at baseline, but were presented in random order. DR grader test scores were compared to baseline and also to those of 29 local ophthalmologists taking part in the NGO DR program, but who did not take the optic nerve grading course.
- Finally, to determine whether further improvement was practical, the non-graders each underwent approximately 20-30 minutes of individually-focused, one-to-one training by a glaucoma specialist (LN), and were tested a third time on the standard image set.

Participants

We enrolled 43 graders currently working in the Orbis and Fred Hollow Foundation (FHF) DR screening project in Vietnam, excluding any ophthalmologists or optometrists. These included nurses, technicians and non-ophthalmic physicians (Table 1). Twenty-nine ophthalmologists in Vietnam from 20 eye hospitals and general hospitals in cities such as Hanoi, Da Nang and Ho Chi Minh City taking part in the project were also recruited for comparison.

The DR graders had already been trained to grade images for DR through the Orbis and FHF DR projects. Participants were informed that if they chose not to participate in the study, their jobs would not be affected in any way.

For peer review only - http://bmjopen.bmj.com/site/about/guidelines.xhtml

Test set of optic nerve images

A total of 60 digital photographic images was used, 33 from eyes with glaucoma and 27 healthy eyes. Among these, 19 images were obtained from the Orbis Vietnam program, 11 from the population-based NICOLA (Northern Ireland Cohort for the Longitudinal Study of Ageing) study and 23 from the Glaucomatous Optic Neuropathy Evaluation (GONE, https://gone-project.com/newgone/) training website. All images were of gradable quality in the opinion of the standard panel of glaucoma specialists, though clarity was not always optimal in order to mimic real world conditions. Images from the GONE website were categorized into difficult, intermediate and easy (Table 1) on the basis of the prior performance of several thousand website users in characterizing them as normal or glaucomatous.[11] The GONE images were graded into normal and abnormal by investigators (NC, AAB, MC, OO, all fellowship-trained glaucoma experts, and TP, a medical retina specialist with a clinical and research focus on image-based screening for eye disease). The images from NICOLA and the Orbis Vietnam program were also sorted into normal and abnormal based on characteristic glaucomatous features of the images by the same group of investigators. When there was internal disagreement between the investigators, the image was replaced with another on which all five agreed.

Although optic nerve images are considered personal data, all images in the test set were fully anonymized and de-identified. Blanket permission for research use of images was obtained through the NICOLA study, Orbis Vietnam DR program and the GONE website, all of which have received prior ethics approval.

Online training course

The online training course was delivered on the Orbis Cybersight website,(cybersight.org) and consisted of three modules (217 Vietnamese-language PowerPoint slides with voice-over narration in Vietnamese) describing a standardized, step-by-step process of evaluating the

For peer review only - http://bmjopen.bmj.com/site/about/guidelines.xhtml

healthy and glaucomatous optic nerve head. Module 1 provided a simple introduction to glaucoma. Module 2 introduced the normal optic nerve head and a systematic description of its features, with some reference to images depicting glaucomatous damage. Module 3 described a systematic method for assessing optic nerve head photos to screen for glaucomatous features using "the 5 Cs": Cup to disc ratio, color of the neuro-retinal rim, comparison between the optic nerve heads in a single patient's two eyes, contour of the nerve, and "concerns" (this includes optic disc haemorrhage, and papilledema, or optic nerve oedema with blurred nerve head margins). At the end of modules 2 and 3, trainees were provided 50 normal optic nerve images and 50 images of glaucomatous nerves for practice, all drawn from the same three sources as the test set (the Orbis Vietnam DR program, the NICOLA study and the GONE website).

Optic nerve photos suggestive of other ocular diseases besides glaucoma were excluded from the course, with the exception of potentially fatal conditions affecting the optic nerve, including optic nerve edema as above. All modules were independently assessed and validated by the five investigators listed above (AAB, MC, TP, NC, OO) for content and appropriateness for these cadres of health workers.

Evaluation with the test set

As above, the set consisted of 60 images, which were presented without stereo on the Orbis Cybersight website, and were each graded as "refer" (likely glaucoma) or "do not refer" (normal) by the test-taker. For images graded as "refer," the correct reason had to be selected from a list. A score of five marks was given for a correct 'no refer' decision. For a correct 'refer' decision, the participant received a score of 5 marks, and an additional 5 marks for every correct reason given for the referral, ranging from one to six reasons. No marks were deducted for incorrect responses. Each of the 60 questions was worth between 5 and 30 marks (depending on the

For peer review only - http://bmjopen.bmj.com/site/about/guidelines.xhtml

number of correct reasons for referral), and a total of 470 marks were available on the test. A percentage score was calculated for each grader as the number of marks received divided by 470.

Correct answers were not revealed to the graders. The questions were presented in a random order, which differed between pre-test and post-test, and also from one test-taker to the other. None of the optic nerve images used in the practice test were included in the quiz.

Additional focused one-to-one training

In order to determine if additional, measurable performance improvements could be realized with further, more intensive training, the DR graders each underwent 20-30 minutes of one-to-one training in optic nerve recognition by a glaucoma specialist (LN) to better distinguish presence of glaucomatous optic nerve damage. The training focused on individual areas of deficiency identified by the post-course training and was conducted online due to Covid-related restrictions on face-to-face gatherings in Vietnam at the time. All 43 (100%) DR graders completed the additional training, and all underwent a final round of testing on the standard image set.

Sample size and statistical methods

The minimum sample size of 42 participants (graders) was calculated using the paired sample size formula for qualitative outcome data reported by Hulley et al.[12] with confidence level of 95% and statistical power of 80%. Using the statistics from the GONE website, we estimated that the mean for our study participants at baseline (pre-training) would be at the 5th percentile in grading performance, while at the end of the course (post-training), they would be at the 50th percentile. Thus, our study was powered to detect an improvement from a score of 29% to 60% correct answers between baseline and post-training performance on the test set.

For peer review only - http://bmjopen.bmj.com/site/about/guidelines.xhtml

True positives (TP) were images correctly graded as glaucomatous optic neuropathy while true negatives (TN) were those correctly graded as 'no glaucoma'. False positive (FP) were images that did not have any features suggestive of glaucomatous optic neuropathy but were incorrectly graded as glaucomatous, while false negatives (FN) were images with glaucomatous features that were incorrectly graded as normal by the test-taker. Sensitivity was then calculated as TP divided by the sum of TP and FN, specificity as TN divided by the sum of TN and FP, positive predictive value (PPV) as TP divided by the sum of TP and FP, and negative predictive value (NPV) as TN divided by the sum of TN and FN. Additionally, area under the receiver operating characteristic (AUC) curve and kappa statistic were computed, with their 95% confidence intervals (95% CI). The performance of DR graders after training was compared to that before training, and also to that of ophthalmologists who had not taken the course.

Since this was a study involving diabetic retinopathy graders and ophthalmologists, we did not involve patients and public in the design or conduct or reporting or dissemination of the study

Results

In total, 43 DR graders participated in the training course and completed both pre and-post training tests, and the additional test after the one-to-one training, while 29 Vietnamese ophthalmologists underwent a single test and did not take part in the training. All DR graders undergoing training completed three rounds of testing.

The mean age (32.6±5.5 years) did not differ from that of the ophthalmologists (32.3±7.3 years, p=0.893, Table 1). There was also no statistically significant difference between the age, sex distribution and work experience of DR graders vs ophthalmologists. The DR graders required a mean of 297.8 minutes (144.6), to complete the course according to measurements carried out automatically by the Orbis Cybersight website.

For peer review only - http://bmjopen.bmj.com/site/about/guidelines.xhtml

DR graders improved significantly between their mean pre-training percent scores (33.3% ± 14.3%) and their post-training performance score [(55.8% ±12.6%), P <0.001)]. (Table 2) The mean post-training score for DR graders did not differ significantly from that for local ophthalmologists (58.7±15.4%, P=0.384]. These findings, significant improvement between pre and post-training tests and the lack of a significant difference between post-training tests by DR graders and the performance of ophthalmologists, were consistent across optic nerve images from all sources. (Table 2)

With respect to other indices of grading accuracy (Table 3), there was no significant change between DR graders' pre-training [85.5% (83.5 – 87.3)] and post-training sensitivity [80.4% (78.3 – 82.4), while specificity improved significantly [47.8 (44.9 – 50.7) to 79.8 (77.3 – 82.0), p<0.001]. The positive and negative predictive values also improved significantly between pre- and post-training tests, as did the area under the ROC curve [66.6 (64.9 – 68.3) to 80.1 (78.5 – 81.6), p<0.001]. (Table 3) When compared to the 29 ophthalmologists, the 43 DR graders after training had worse sensitivity, better specificity, worse negative predictive value and worse kappa. The positive predictive value and the area under the ROC curve (our pre-specified indicator of overall grading accuracy) did not differ between post-training DR graders and ophthalmologists. (Table 3)

Table 4 shows the predictors of the percentage of correct responses among study participants. In the univariate analysis, age <30 years (p=0.005) and experience < 5 years (p<0.001) predicted higher scores. However, in the multivariate analysis, only experience < 5 years remained significant (p=0.006)**.** The difference between post-training graders and local ophthalmologists remained non-significant when adjusting for other factors.

The test performance of DR graders (n=43) did not improve significantly after additional focused, one-to-one training, when compared to their performance after the online training.

For peer review only - http://bmjopen.bmj.com/site/about/guidelines.xhtml

(Table 5) Their mean scores still did not differ from the ophthalmologists after this additional focused training. These patterns were the same for optic nerve images from all sources

For peer review only

For peer review only - http://bmjopen.bmj.com/site/about/guidelines.xhtml

Discussion

This uncontrolled, experimental, before-and-after study found a significant improvement in the baseline mean scores of non-ophthalmic DR graders after undergoing a 5-hour training course. The post course scores of the DR graders were almost double the baseline scores, and did not differ significantly from the scores of general ophthalmologists in Vietnam. This suggests that it is possible to train non ophthalmic DR graders to screen accurately for glaucoma using optic nerve photos using a short, self-paced online course. Additional, more intensive one-to-one training by an expert did not result in significant further improvement of grading performance, suggesting encouragingly that results obtained with a convenient and scalable tool like our course approach is what may be readily achievable with such cadres.

In many developing countries, half or more of glaucoma patients present with advanced disease.[13–16] Shortage of providers limits access to eye care and leads to delays in diagnosis and management of glaucoma until late in the disease process, often too late for effective, sight-saving treatment.[17,18] For example, a recent study reported > 60% prevalence of blindness in the operative eye among patients at the time of first glaucoma surgery in rural China[19] .Training non-ophthalmic graders to screen for glaucoma may be an initial step in promoting earlier glaucoma diagnosis in low resource settings. This has been described as task shifting, a model in which lower cadres of staff are trained to do simpler tasks so that scarcer and more highly-trained workers such as ophthalmologists can concentrate on high-level medical decision making and surgical management.[20-23]

The graders participating in this study were already trained to detect DR, the most frequent microvascular complication of diabetes and the leading cause of blindness in working-age adults. Studies[24–26] have demonstrated that > 90% of severe vision loss from DR can be prevented through regular screening and prompt referral and treatment. Diabetic screening is more cost-effective compared to no screening or opportunistic screening.[27,28] Glaucoma is the

For peer review only - http://bmjopen.bmj.com/site/about/guidelines.xhtml

most common cause of irreversible blindness globally[2,29] and glaucoma blindness can similarly be prevented by regular screening of high-risk persons, prompt referral and treatment. Training the same cadres to screen for these two major causes of blindness may further improve cost effectiveness and cost-benefit of screening. This model is of particular interest in low-income settings, where traditional eye health services are mainly located in urban centres, often with poor accessibility to rural populations.

The model assessed in the current study differs from that described in previous reports in several ways. Non-expert health workers were trained to recognize glaucoma using an online course, and their performance was objectively measured and compared to their baseline, and to the scores of local ophthalmologists who had not taken the course. Previous studies have used telemedicine to send retinal images to reading centres for expert grading.[30–32] This can be challenging to sustain in low-resource settings, as it still depends on the time and availability of ophthalmologists or other relatively scarce trained experts to review retinal images for evidence of disease. Reading centres can also be logistically complex and expensive to establish and maintain in low resource settings, due to challenges such as the low bandwidth of available internet connections.

An advantage of training non experts as in our current model is that they are more likely to be able to review the images in real time due to lack of competing clinical duties. In delayed grading models, such as those using telemedicine, patients have to be contacted later to notify them of their screening results. Existing studies in LMICs have shown that this can lead to substantial loss to follow-up.[33] The RAIDERS study in Rwanda reported that patients receiving immediate feedback on the need for referral demonstrated higher adherence with recommended services compared to participants informed of the results of their screening several days later.[33]

For peer review only - http://bmjopen.bmj.com/site/about/guidelines.xhtml

Training with our course significantly improved graders' specificity and positive predictive value. This effect of training is particularly relevant in low-resource settings, where the priority must be to avoid over-burdening already weak health systems with large numbers of false positive referrals. Ausayakhun et al[34] described a somewhat comparable model to ours, of training non-ophthalmologists to deliver point-of-service screening for glaucoma, cataract and age-related macular degeneration, and their reported specificity for glaucoma was approximately 80% using fundus photographs, similar to that observed in the current study.

An important determinant of the positive predictive value of a screening program is the burden of disease in the target population.[35] The heavy burden of glaucoma among Asians[36–38] and Africans[39–41] has been well-documented. It is estimated that Asia has the highest number of persons living with glaucoma globally, and that this figure will increase by 79.8% (primary open-angle glaucoma) and 58.4% (primary angle closure glaucoma) by 2040.[3] In view of this high glaucoma burden in Asia, and the growing gap between need and supply for ophthalmologists globally,[42] it is clear that new screening models are needed which rely on non-experts. We were able to train diabetic graders to perform at the level of local ophthalmologists, suggesting that our training course can deliver successful and effective task shifting.

An important predictor of correct responses on the test set among study participants was working experience of < 5 years. Lack of a positive effect of cumulative experience may seem counter-intuitive, but we suggest this may reflect better performance among younger and more-recently-trained cadres. Further studies are needed to replicate and better understand this finding.

Although artificial intelligence (AI) has been used successfully for DR screening,[43,44] well trained human graders are crucial in the validation of these AI systems, which must employ locally-derived images and personnel to be most reliable. Moreover, there are substantial existing legal

For peer review only - http://bmjopen.bmj.com/site/about/guidelines.xhtml

and financial barriers to widespread sustainable use of AI for DR and glaucoma screening in many LMICs. Human systems are needed until these are overcome.

Strengths of this study include an approach to assessing the impact of training which included both a before-after comparison and external validation against the performance of local ophthalmologists. Success of the course against both of these criteria was consistent across images of varying difficulty and drawn from a wide variety of sources. Both the course and the assessment test reflected input from clinical experts, specialists in image-based screening and those engaged professionally in capacity-building for the recognition of glaucomatous optic nerves. A number of widely-used indices of screening accuracy were computed, allowing us to elucidate specific benefits of training in improving specificity.

This study has limitations as well. The generalizability of our results to other cadres of trainees and other settings is uncertain. Most importantly, further research is needed to better understand the practicality of this training model within the context of a full glaucoma screening program. Additional work is under way to test the model as part of delivering outreach screening for glaucoma in sub-Saharan Africa, aimed at a broad variety of patients at risk for glaucoma, and not solely those with DR.

In summary, our study shows that it is possible to train non ophthalmic DR graders quickly and at low cost to screen for glaucoma using optic nerve images. This model may improve glaucoma detection and ultimately reduce the prevalence of blindness from glaucoma in low resource settings.

For peer review only

For peer review only - http://bmjopen.bmj.com/site/about/guidelines.xhtml

Table 1: Characteristics of DR graders and ophthalmologists participating in a study of glaucomatous optic nerve grading

Characteristics	Ophthalmologists (n=29)	DR graders (n=43)	T-test/Chi-Square (P-value)
**Age (Mean±SD) yrs**	32.6±5.5	32.3±7.3	0.13 (0.893)
<30 years	11 (37.9%)	21 (48.8%)	0.83 (0.361)
≥30 years	18 (62.1%)	22 (51.2%)	
**Sex**			1.99 (0.159)
Female (%)	17 (58.6%)	32 (74.4%)	
Male (%)	12 (41.4%)	11 (25.6%)	
**Job Title**			
Nurse (%)	0 (0.0%)	18 (41.9%)	
Ophthalmologist (%)	29 (100.0%)	0 (0.0%)	
Other physician (%)*	0 (0.0%)	22 (51.2%)	
Technician (%)	0 (0.0%)	3 (6.9%)	
**Work Experience (Mean±SD) yrs**	7.21±5.2	8.22±7.1	0.65 (0.516)
<5 (%)	11 (37.9%)	18 (41.9%)	0.11 (0.739)
≥5 (%)	18 (62.1%)	25 (58.1%)	

*Endocrinologist (5); General Practitioner (14); Radiologist (2); Internist (1)

For peer review only

Table 2: Accuracy (% Correct vs Gold Standard*) in grading optic nerve images from different sources, comparing DR graders and ophthalmologists

Source of Images	Number of images	% Pre-training Score of graders (A) Mean±SD	% Post-training Score of graders (B) Mean±SD	P-value) (A vs B)	% Scores of Ophthalmologists (C) Mean±SD	P-value (B vs C)
GONE Website						
Easy	8	31.9±17.6	59.2±17.6	<0.001	65.1±21.9	0.216
Moderate	8	25.6±13.3	44.2±19.4	<0.001	48.1±17.8	0.392
Hard	7	22.7±15.9	47.7±22.5	<0.001	46.2±21.8	0.780
Orbis Vietnam DR program	19	47.0±22.7	70.3±12.0	<0.001	70.3±18.2	0.991
NICOLA population-based study in Northern Ireland	11	31.4±20.0	58.7±16.9	<0.001	55.2±22.1	0.443
Other images illustrating specific findings	4	31.0±19.8	51.9±24.1	<0.001	57.5±23.4	0.337
****Total**	60	33.3± 14.3	55.8±12.6	<0.001	58.7±15.4	0.384

*A panel of four fellowship-trained glaucoma specialist ophthalmologists and one medical retina specialist with a clinical and research focus on image-based screening for eye disease (AAB, MC, TP, NC, OO) **The remaining 3 images came from a variety of other sources.

For peer review only - http://bmjopen.bmj.com/site/about/guidelines.xhtml

Table 3: Accuracy in grading optic nerve images, comparing DR graders and ophthalmologists, and calculated against a panel of specialist ophthalmologists (the “gold standard”).

Participant Type	Sensitivity (95%CI)	Specificity (95%CI)	Positive Predictive Value (95%CI)	Negative Predictive Value (95%CI)	Kappa (95%CI)	Area under the Receiver-Operator Curve (95%CI)
DR graders, Pre-Training	85.5 (83.5 – 87.3)	47.8 (44.9 – 50.7)	66.7 (65.4 – 68.0)	72.9 (70.1 – 75.6)	34.4 (30.9 – 37.9)	66.6 (64.9 – 68.3)
DR graders, Post-training	80.4 (78.3 – 82.4)	79.8 (77.3 – 82.0)	82.9 (81.2 – 84.5)	76.9 (74.9 – 78.8)	60.0 (56.9 – 63.1)	80.1 (78.5 – 81.6)
P-value Comparing Graders Pre and Post-training	<0.05	<0.05	<0.05	<0.05	<0.05	<0.001
Ophthalmologists (No Training Given)	92.9 (91.1 – 94.4)	73.8 (70.6 – 76.9)	81.3 (79.4 – 83.0)	89.5 (87.1 – 91.5)	67.8 (64.3 – 71.3)	83.4 (81.6 – 85.1)
P-value comparing Post-training graders and Ophthalmologists	<0.05	<0.05	Not significant	<0.05	<0.05	Not significant

For peer review only

Table 4: Predictors of the percentage of correct responses on the test set among study participants

Potential Predictor	Univariate Analysis			Multivariate Analysis		
	Beta	95% Confidence Interval	P-value	Beta	95% Confidence Interval	P-value
Age <30 years	9.0	2.8 – 15.2	0.005	0.4	-8.7 – 9.4	0.938
Male sex	0.4	-6.5 – 7.4	0.899	1.8	-4.7 – 8.3	0.585
Post-training non-ophthalmic grader (Vs ophthalmologist)	-2.9	-9.5 – 3.7	0.384	-3.1	-9.2 – 3.1	0.319
<5 years of work experience	12.5	6.6 – 18.4	<0.001	13.1	3.9 – 22.3	0.006
Nurse (vs "Other physicians," non-ophthalmic grader group only)	-4.5	12.3 – 3.3	0.251			
Pre vs post-training (DR graders only)	0.4	0.1 – 0.6	0.007			

For peer review only

For peer review only - http://bmjopen.bmj.com/site/about/guidelines.xhtml

Table 5: Accuracy (% Correct vs Gold Standard*) in grading optic nerve images from different sources: the effect of additional one-one training for DR graders after the online course

Source of Images	Number of images	% Post-training score of graders after online course Mean±SD (A) (n=43)	% Post training score of graders after additional focused one-one training, Mean±SD (B) (n=43)	P-value) (A vs B)	% Scores of Ophthalmologists Mean±SD (C) (n=29)	P-value (B vs C)
GONE Website						
Easy	8	59.2±17.6	60.0±21.6	0.9364	65.1±21.9	0.6463
Moderate	8	44.2±19.4	48.7±21.9	0.6702	48.1±17.8	0.9529
Hard	7	47.7±22.5	49.7±25.8	0.8797	46.2±21.8	0.7886
Orbis Vietnam DR program	19	70.3±12.0	69.8±14.8	0.9096	70.3±18.2	0.9265
NICOLA population-based study in Northern Ireland	11	58.7±16.9	66.7±14.8	0.2514	55.2±22.1	0.1670
Other images illustrating specific findings	4	51.9±24.1	56.1±26.4	0.8220	57.5±23.4	0.9393
****Total**	**60**	**55.8±12.6**	**60.3±16.4**	**0.1573**	**58.7±15.4**	**0.6422**

For peer review only - http://bmjopen.bmj.com/site/about/guidelines.xhtml

Ethics statement

Approval for the study was obtained from the Ethical Review Board of the Queen's University Belfast United Kingdom (MHLS 20_98), and the Ethics Committee of the Hanoi Medical College in Vietnam (IRB-VN01.001/IRB 00003121/ FWA 00004148, Approval number 587)

For peer review only

For peer review only - http://bmjopen.bmj.com/site/about/guidelines.xhtml

References

1. Flaxman SR, Bourne RRA, Resnikoff S, et al. Global causes of blindness and distance vision impairment 1990-2020: a systematic review and meta-analysis. *Lancet Glob Heal*. 2017;5(12):e1221-e1234. doi:10.1016/S2214-109X(17)30393-5

2. Causes of blindness and vision impairment in 2020 and trends over 30 years, and prevalence of avoidable blindness in relation to VISION 2020: the Right to Sight: an analysis for the Global Burden of Disease Study. *Lancet Glob Heal*. 2021;9(2):e144-e160. doi:10.1016/S2214-109X(20)30489-7

3. Tham YC, Li X, Wong TY, Quigley HA, Aung T, Cheng CY. Global prevalence of glaucoma and projections of glaucoma burden through 2040: A systematic review and meta-analysis. *Ophthalmology*. 2014;121(11). doi:10.1016/j.ophtha.2014.05.013

4. Bourne RRA, Taylor HR, Flaxman SR, et al. Number of people blind or visually impaired by glaucoma worldwide and in world regions 1990 - 2010: A meta-analysis. *PLoS One*. 2016;11(10):1-16. doi:10.1371/journal.pone.0162229

5. Dandona R, Dandona L, Naduvilath TJ, McCarty CA, Rao GN. Utilisation of eyecare services in an urban population in southern India: the Andhra Pradesh eye disease study. *Br J Ophthalmol*. 2000;84(1):22-27. doi:10.1136/bjo.84.1.22

6. Ramakrishnan R, Nirmalan PK, Krishnadas R, et al. Glaucoma in a rural population of Southern India: The Aravind Comprehensive Eye Survey. *Ophthalmology*. 2003;110(8):1484-1490. doi:10.1016/S0161-6420(03)00564-5

7. Burr JM, Mowatt G, Hernández R, et al. The clinical effectiveness and cost-effectiveness of screening for open angle glaucoma: A systematic review and economic evaluation. *Health Technol Assess (Rockv)*. 2007;11(41). doi:10.3310/hta11410

8. Hernandez R, Rabindranath K, Fraser C, Vale L, Blanco AA, Burr JM. Screening for open angle glaucoma: systematic review of cost-effectiveness studies. *J Glaucoma*. 2008;17(3):159-168. doi:10.1097/IJG.0b013e31814b9693

9. Tang J, Liang Y, O'Neill C, Kee F, Jiang J, Congdon N. Cost-effectiveness and cost-utility of population-based glaucoma screening in China: a decision-analytic Markov model. *Lancet Glob Heal*. 2019;7(7):e968-e978. doi:10.1016/S2214-109X(19)30201-3

10. John D, Parikh R. Cost-effectiveness of community screening for glaucoma in rural India: a decision analytical model. *Public Health*. 2018;155:142-151. doi:10.1016/j.puhe.2017.11.004

11. Kong YX, Coote MA, O'Neill EC, Gurria LU, Xie J, Garway-Heath D, Medeiros FA, Crowston JG. Glaucomatous optic neuropathy evaluation project: a standardized internet system for assessing skills in optic disc examination. Clin Exp Ophthalmol. 2011 May-Jun;39(4):308-17. doi: 10.1111/j.1442-9071.2010.02462.x. Epub 2011 Jan 14. PMID: 21070546.

12. Hulley S. *Designing Clinical Research (3rd Ed.) Philadeiphia, PA: Lippioncott Williams, Wilkins*.; 2007.

13. Olawoye O, Kizor-Akaraiwe N, Pons J, et al. Clinical Characteristics and Stage at Presentation of Glaucoma Patients in Sub-Saharan Africa. *J Glaucoma*. June 2022. doi:10.1097/IJG.0000000000002068

14. Gyasi ME, Francis AW, Chen Y, Harrison RSR, Kodjo AR. Presentation of glaucoma in the greater Accra metropolitan area of Ghana. *Ghana Med J*. 2014;48(3):143-147.

For peer review only - http://bmjopen.bmj.com/site/about/guidelines.xhtml

15. Omoti AE, Osahon AI, Waziri-Erameh MJM. Pattern of presentation of primary open-angle glaucoma in Benin City, Nigeria. *Trop Doct*. 2006;36(2):97-100. doi:10.1258/004947506776593323

16. Abdull MM, Gilbert CC, Evans J. Primary open angle glaucoma in northern Nigeria: stage at presentation and acceptance of treatment. *BMC Ophthalmol*. 2015. doi:10.1186/s12886-015-0097-9

17. Mansberger SL, Sheppler C, Barker G, et al. Long-term Comparative Effectiveness of Telemedicine in Providing Diabetic Retinopathy Screening Examinations: A Randomized Clinical Trial. *JAMA Ophthalmol*. 2015;133(5):518-525. doi:10.1001/jamaophthalmol.2015.1

18. Estopinal CB, Ausayakhun S, Ausayakhun S, et al. Access to ophthalmologic care in Thailand: a regional analysis. *Ophthalmic Epidemiol*. 2013;20(5):267-273. doi:10.3109/09286586.2013.821498

19 Yang K, Jin L, Li L*, et al* Preoperative characteristics and compliance with follow-up after trabeculectomy surgery in rural southern China. *British Journal of Ophthalmology* 2017;**101:**131-137.

20. Gichangi M, Kalua K, Barassa E, Eliah E, Lewallen S, Courtright P. Task Shifting for Eye Care in Eastern Africa: General Nurses as Trichiasis Surgeons in Kenya, Malawi, and Tanzania. *Ophthalmic Epidemiol*. 2015;22(3):226-230. doi:10.3109/09286586.2015.1040924

21 Bascaran C, Mwangi N, D'Esposito F, et al. Effectiveness of task-shifting for the detection of diabetic retinopathy in low- and middle-income countries: a rapid review protocol. *Syst Rev*. 2021;10(1):4. doi:10.1186/s13643-020-01553-

22 Shah M, Noor A, Deverell L, Ormsby GM, Harper CA, Keeffe JE. Task sharing in the eye care workforce: Screening, detection, and management of diabetic retinopathy in Pakistan. A case study. *Int J Health Plann Manage*. March 2018. doi:10.1002/hpm.2508

23. Bolme S, Morken TS, Follestad T, Sørensen TL, Austeng D. Task shifting of intraocular injections from physicians to nurses: a randomized single-masked noninferiority study. *Acta Ophthalmol*. 2020;98(2):139-144. doi:10.1111/aos.14184

24. Swanson M. Retinopathy screening in individuals with type 2 diabetes: who, how, how often, and at what cost--an epidemiologic review. *Optometry*. 2005;76(11):636-646. doi:10.1016/j.optm.2005.08.019

25. Cheung N, Mitchell P, Wong TY. Diabetic retinopathy. *Lancet*. 2010;376(9735):124-136. doi:10.1016/S0140-6736(09)62124-3

26. Stewart MW. Treatment of diabetic retinopathy: Recent advances and unresolved challenges. *World J Diabetes*. 2016;7(16):333-341. doi:10.4239/wjd.v7.i16.333

27. Mead A, Burnett S, Davey C. Diabetic retinal screening in the UK. *J R Soc Med*. 2001;94(3):127-129. doi:10.1177/014107680109400307

28. Jones S, Edwards RT. Diabetic retinopathy screening: a systematic review of the economic evidence. *Diabet Med*. 2010;27(3):249-256. doi:10.1111/j.1464-5491.2009.02870.x

29. Quigley HA, Broman AT. The number of people with glaucoma worldwide in 2010 and

For peer review only - http://bmjopen.bmj.com/site/about/guidelines.xhtml

2020. *Br J Ophthalmol*. 2006;90(3):262-267. doi:10.1136/bjo.2005.081224

30. Rathi S, Tsui E, Mehta N, Zahid S, Schuman JS. The Current State of Teleophthalmology in the United States. *Ophthalmology*. 2017;124(12):1729-1734. doi:10.1016/j.ophtha.2017.05.026

31. Hark LA, Katz LJ, Myers JS, et al. Philadelphia Telemedicine Glaucoma Detection and Follow-up Study: Methods and Screening Results. *Am J Ophthalmol*. 2017;181:114-124. doi:10.1016/j.ajo.2017.06.024

32. Joseph S, Kim R, Ravindran RD, Fletcher AE, Ravilla TD. Effectiveness of Teleretinal Imaging-Based Hospital Referral Compared With Universal Referral in Identifying Diabetic Retinopathy: A Cluster Randomized Clinical Trial. *JAMA Ophthalmol*. 2019;137(7):786-792. doi:10.1001/jamaophthalmol.2019.1070

33 Mathenge W, Whitestone N, Nkurikiye J, et al. Impact of Artificial Intelligence Assessment of Diabetic Retinopathy on Referral Service Uptake in a Low-Resource Setting: The RAIDERS Randomized Trial. *Ophthalmol Sci*. 2022;2(4):100168. doi:https://doi.org/10.1016/j.xops.2022.100168

34. Ausayakhun S, Snyder BM, Ausayakhun S, et al. Clinic-Based Eye Disease Screening Using Non-Expert Fundus Photo Graders at the Point of Screening: Diagnostic Validity and Yield. *Am J Ophthalmol*. 2021;227:245-253. doi:10.1016/j.ajo.2021.03.029

35. Chasan JE, Delaune B, Maa AY, Lynch MG. Effect of a teleretinal screening program on eye care use and resources. *JAMA Ophthalmol*. 2014;132(9):1045-1051. doi:10.1001/jamaophthalmol.2014.1051

36. Wang YX, Xu L, Yang H, Jonas JB. Prevalence of glaucoma in North China: the Beijing Eye Study. *Am J Ophthalmol*. 2010;150(6):917-924. doi:10.1016/j.ajo.2010.06.037

37. Casson RJ, Newland HS, Muecke J, et al. Prevalence of glaucoma in rural Myanmar: the Meiktila Eye Study. *Br J Ophthalmol*. 2007;91(6):710-714. doi:10.1136/bjo.2006.107573

38. Song W, Shan L, Cheng F, et al. Prevalence of glaucoma in a rural northern china adult population: a population-based survey in kailu county, inner mongolia. *Ophthalmology*. 2011;118(10):1982-1988. doi:10.1016/j.ophtha.2011.02.050

39. Budenz DL, Barton K, Whiteside-de Vos J, et al. Prevalence of glaucoma in an urban West African population: the Tema Eye Survey. *JAMA Ophthalmol*. 2013;131(5):651-658. doi:10.1001/jamaophthalmol.2013.1686

40. Ashaye A, Ashaolu O, Komolafe O, et al. Prevalence and types of glaucoma among an indigenous African population in southwestern Nigeria. *Investig Ophthalmol & Vis Sci*. 2013;54(12).

41. Rotchford AP, Kirwan JF, Muller MA, Johnson GJ, Roux P. Temba glaucoma study: a population-based cross-sectional survey in urban South Africa. *Ophthalmology*. 2003;110(2):376-382. doi:10.1016/S0161-6420(02)01568-3

42 Resnikoff S, Felch W, Gauthier TM, Spivey B. The number of ophthalmologists in practice and training worldwide: a growing gap despite more than 200,000 practitioners. Br J Ophthalmol. 2012 Jun;96(6):783-7. doi: 10.1136/bjophthalmol-2011-301378. Epub 2012 Mar 26. PMID: 22452836.

43 Bellemo V, Lim ZW, Lim G, Nguyen QD, Xie Y, Yip MYT, Hamzah H, Ho J, Lee XQ, Hsu W, Lee ML, Musonda L, Chandran M, Chipalo-Mutati G, Muma M, Tan GSW, Sivaprasad

For peer review only - http://bmjopen.bmj.com/site/about/guidelines.xhtml

S, Menon G, Wong TY, Ting DSW. Artificial intelligence using deep learning to screen for referable and vision-threatening diabetic retinopathy in Africa: a clinical validation study. Lancet Digit Health. 2019 May;1(1):e35-e44. doi: 10.1016/S2589-7500(19)30004-4. Epub 2019 May 2. PMID: 33323239.

44 Hansen MB, Abràmoff MD, Folk JC, Mathenge W, Bastawrous A, Peto T (2015) Results of Automated Retinal Image Analysis for Detection of Diabetic Retinopathy from the Nakuru Study, Kenya. PLoS ONE 10(10): e0139148. https://doi.org/10.1371/journal.pone.0139148

For peer review only

STARD Checklist

Section & Topic	No	Item	Reported on page #
**TITLE OR ABSTRACT**			
	**1**	Identification as a study of diagnostic accuracy using at least one measure of accuracy (such as sensitivity, specificity, predictive values, or AUC)	Page 2
**ABSTRACT**			
	**2**	Structured summary of study design, methods, results, and conclusions (for specific guidance, see STARD for Abstracts)	Page 2
**INTRODUCTION**			
	**3**	Scientific and clinical background, including the intended use and clinical role of the index test	Page 5 and 6
	**4**	Study objectives and hypotheses	Page 6
**METHODS**			
Study design	**5**	Whether data collection was planned before the index test and reference standard were performed (prospective study) or after (retrospective study)	Page 6 and 7
Participants	**6**	Eligibility criteria	Page 7
	**7**	On what basis potentially eligible participants were identified (such as symptoms, results from previous tests, inclusion in registry)	Page 7
	**8**	Where and when potentially eligible participants were identified (setting, location and dates)	Page 7
	**9**	Whether participants formed a consecutive, random or convenience series	Page 7
Test methods	**10a**	Index test, in sufficient detail to allow replication	Pages 8-10
	**10b**	Reference standard, in sufficient detail to allow replication	Pages 9-10
	**11**	Rationale for choosing the reference standard (if alternatives exist)	
	**12a**	Definition of and rationale for test positivity cut-offs or result categories of the index test, distinguishing pre-specified from exploratory	Page 10-11
	**12b**	Definition of and rationale for test positivity cut-offs or result categories of the reference standard, distinguishing pre-specified from exploratory	
	**13a**	Whether clinical information and reference standard results were available to the performers/readers of the index test	NA
	**13b**	Whether clinical information and index test results were available to the assessors of the reference standard	9-11
Analysis	**14**	Methods for estimating or comparing measures of diagnostic accuracy	10-11
	**15**	How indeterminate index test or reference standard results were handled	NA
	**16**	How missing data on the index test and reference standard were handled	NA
	**17**	Any analyses of variability in diagnostic accuracy, distinguishing pre-specified from exploratory	
	**18**	Intended sample size and how it was determined	10-11
**RESULTS**			
Participants	**19**	Flow of participants, using a diagram	No diagram but well described on page 7
	**20**	Baseline demographic and clinical characteristics of participants	Page 11, table 1
	**21a**	Distribution of severity of disease in those with the target condition	NA
	**21b**	Distribution of alternative diagnoses in those without the target condition	NA
	**22**	Time interval and any clinical interventions between index test and reference standard	Table 4
Test results	**23**	Cross tabulation of the index test results (or their distribution) by the results of the reference standard	Table 2
	**24**	Estimates of diagnostic accuracy and their precision (such as 95% confidence intervals)	Table 3
	**25**	Any adverse events from performing the index test or the reference standard	NA
**DISCUSSION**			
	**26**	Study limitations, including sources of potential bias, statistical uncertainty, and generalisability	Page 17

For peer review only

For peer review only - http://bmjopen.bmj.com/site/about/guidelines.xhtml

	**27**	Implications for practice, including the intended use and clinical role of the index test	Pages 16 -17
**OTHER INFORMATION**			
	**28**	Registration number and name of registry	NCT05151757 ClinicalTrials.gov
	**29**	Where the full study protocol can be accessed	
	**30**	Sources of funding and other support; role of funders	eXcellence in Ophthalmology and Vision Awards (XOVA Novartis)

For peer review only

For peer review only

BMJ Open

Impact of a short online course on the accuracy of non-ophthalmic diabetic retinopathy graders in recognizing glaucomatous optic nerves in Vietnam

Journal:	*BMJ Open*
Manuscript ID	bmjopen-2023-076623.R1
Article Type:	Original research
Date Submitted by the Author:	21-Aug-2023
Complete List of Authors:	Olawoye, Olusola; Queens University Belfast, Dentistry and Biomedical Sciences; University of Ibadan College of Medicine, Ophthalmology<br>Ha, Thu Huong; ORBIS International<br>Pham, Ngoc; ORBIS International<br>Nguyen, Lam; Hanoi Medical University<br>Cherwek, David; ORBIS International, Global Programs<br>Fowobaje, Kayode Raphael; University of Ibadan, Department of Epidemiology and Medical Statistics<br>Ross, Craig; Centre for Eye Research Australia<br>Coote, Michael; Centre for Eye Research Australia<br>Chan, Ving Fai; Queens University Belfast, Dentistry and Biomedical Sciences<br>Kahook, Malik; University of Colorado at Colorado Springs<br>Peto, Tunde; Queen's University Belfast Faculty of Medicine Health and Life Sciences<br>Azuara-Blanco, Augusto ; Queens University Belfast , Centre for Public Health<br>Congdon, Nathan; Queen's University Belfast, Ophthalmology and Public Health
**Primary Subject Heading**:	Ophthalmology
Secondary Subject Heading:	Epidemiology, Global health
Keywords:	Glaucoma < OPHTHALMOLOGY, EPIDEMIOLOGY, PUBLIC HEALTH, EPIDEMIOLOGIC STUDIES

SCHOLARONE™
Manuscripts

I, the Submitting Author has the right to grant and does grant on behalf of all authors of the Work (as defined in the below author licence), an exclusive licence and/or a non-exclusive licence for contributions from authors who are: i) UK Crown employees; ii) where BMJ has agreed a CC-BY licence shall apply, and/or iii) in accordance with the terms applicable for US Federal Government officers or employees acting as part of their official duties; on a worldwide, perpetual, irrevocable, royalty-free basis to BMJ Publishing Group Ltd ("BMJ") its licensees and where the relevant Journal is co-owned by BMJ to the co-owners of the Journal, to publish the Work in this journal and any other BMJ products and to exploit all rights, as set out in our licence.

The Submitting Author accepts and understands that any supply made under these terms is made by BMJ to the Submitting Author unless you are acting as an employee on behalf of your employer or a postgraduate student of an affiliated institution which is paying any applicable article publishing charge ("APC") for Open Access articles. Where the Submitting Author wishes to make the Work available on an Open Access basis (and intends to pay the relevant APC), the terms of reuse of such Open Access shall be governed by a Creative Commons licence – details of these licences and which Creative Commons licence will apply to this Work are set out in our licence referred to above.

Other than as permitted in any relevant BMJ Author's Self Archiving Policies, I confirm this Work has not been accepted for publication elsewhere, is not being considered for publication elsewhere and does not duplicate material already published. I confirm all authors consent to publication of this Work and authorise the granting of this licence.

For peer review only

Title: Impact of a short online course on the accuracy of non-ophthalmic diabetic retinopathy graders in recognizing glaucomatous optic nerves in Vietnam

Authors: Olusola Olawoye,[1,2] Thu Huong Ha,[3] Ngoc Pham,[3] Lam Nguyen,[4] David Hunter Cherwek,[5] Kayode Raphael Fowobaje,[6] Craig Ross, [7] Michael Coote,[7] Ving Fai Chan,[1] Malik Y. Kahook,[8] Tunde Peto,[1] Augusto Azuara-Blanco,[1] Nathan Congdon.[1,5,9]

Affiliations:

[1]Centre for Public Health, School of Medicine, Dentistry and Biomedical Sciences. Queens University Belfast, United Kingdom

[2]College of Medicine University of Ibadan Nigeria

[3]Orbis International, Vietnam

[4]Hanoi Medical University, Vietnam

[5] Orbis International, NY, United States

[6]Department of Epidemiology and Medical Statistics, Faculty of Public Health, College of Medicine, University of Ibadan, Nigeria

[7]Centre for Eye Research Australia, East Melbourne, Australia

[8]Sue Anschutz-Rodgers Eye Centre, University of Colorado School of Medicine

[9]Zhongshan Ophthalmic Centre, Sun Yat-sen University, Guangzhou, China

Running Title: Online training for non-ophthalmologists to recognize glaucoma

Corresponding Author: Nathan Congdon

Address: Centre for Public Health, School of Medicine, Dentistry and Biomedical Sciences. Queen's University Belfast, United Kingdom E-mail: ncongdon1@gmail.com

Keywords: Glaucoma, screening, online course, diabetic retinopathy grading, non-ophthalmic graders, training

For peer review only

Abstract

Purpose:

To test an online training course for non-ophthalmic diabetic retinopathy (DR) graders for recognition of glaucomatous optic nerves in Vietnam.

Methods:

This was an uncontrolled, experimental, before-and-after study in which 43 non-ophthalmic DR graders underwent baseline testing on a standard image set, completed a self-paced, online training course and were retested using the same photographs presented randomly. 29 local ophthalmologists completed the same test without the training course. DR graders then underwent additional one-one training by a glaucoma specialist and were re-tested. Test performance (% correct, compared to consensus grades from 4 fellowship-trained glaucoma experts), sensitivity, specificity, positive and negative predictive value, and area under the receiver operating (AUC) curve, were computed.

Results:

Mean age of DR graders (32.6±5.5 years) did not differ from ophthalmologists (32.3±7.3 years, p=0.13). Online training required a mean of 297.9 (SD 144.6) minutes. Graders' mean baseline score (33.3% ± 14.3%) improved significantly after training (55.8% ±12.6%, P <0.001), and post-training score did not differ from ophthalmologists (58.7±15.4%, P=0.384]. Grader sensitivity did not differ before [85.5% (95% CI 83.5 – 87.3)] vs after [80.4% (78.3 – 82.4)] training, while specificity improved significantly [47.8 (44.9 – 50.7) vs 79.8 (77.3 – 82.0), p<0.001]. Grader AUC also improved after training [66.6 (64.9 – 68.3) to 80.1 (78.5 – 81.6), p<0.001]. Additional one-one grader training by a glaucoma specialist did not further improve grader scores.

For peer review only - http://bmjopen.bmj.com/site/about/guidelines.xhtml

Conclusion:

Non-ophthalmic DR graders can be trained to recognise glaucoma using a short online course in this setting, with no additional benefit from more expensive one-one training. After 5-hour online training in recognizing glaucomatous discs, scores of non-ophthalmic diabetic retinopathy graders doubled, and did not differ from local ophthalmologists. Intensive one-to-one training did not further improve performance

For peer review only

For peer review only - http://bmjopen.bmj.com/site/about/guidelines.xhtml

Strengths and Limitations of this study

Strengths

- Use of an approach to assess the impact of training which included both a before-after comparison and external validation against the performance of local ophthalmologists.
- Both the course and the assessment test reflected input from clinical experts, specialists in image-based screening and those engaged professionally in capacity-building for the recognition of glaucomatous optic nerves.
- A number of widely-used indices of screening accuracy were computed, allowing the identification of specific benefits of training in improving specificity.

Limitations

- The generalizability of our results to other cadres of trainees and other settings is uncertain.
- Further research is needed to better understand the practicality of this training model within the context of a full glaucoma screening program.

For peer review only - http://bmjopen.bmj.com/site/about/guidelines.xhtml

Introduction

Glaucoma is the leading cause of irreversible blindness globally. [1,2] The number of persons living with glaucoma has increased from 64.3 million in 2013 to 76.0 million in 2020, [3] and is projected to rise a further 74% by 2040 due to an ageing and growing global population. Asia accounts for 60% of the world's total glaucoma cases, followed by Africa at 13%. [3] The number of persons blind due to glaucoma increased by 0.8 million between 1990 and 2010, with most of this rise occurring in low and middle-income countries (LMICs).[4] This trend is likely to continue without new and more effective screening strategies appropriate for low-resource settings.

Glaucoma often progresses unnoticed until central visual acuity is permanently compromised, therefore early detection of the disease, before symptoms develop is key in reducing the impact of visual loss in these patients. The rate of undiagnosed glaucoma is high, especially in LMICs, where approximately 95% of those affected are not aware of having the disease. [5,6]

Screening of any disease is a form of secondary prevention, with the goal of preventing undesirable outcomes. In the context of glaucoma, screening is designed to promote diagnosis, and treatment if necessary, during the asymptomatic stage in order to prevent further vision loss and blindness. So far, glaucoma screening has not been found to be cost effective in high income countries. [7,8]

Recently, however, a decision-analytic Markov model has been used to show that population screening for both primary open angle glaucoma (POAG) and primary angle closure glaucoma (PACG) was cost effective in both rural and urban China. [9] John et al [10] reported that community screening for glaucoma in India would prevent 2,190 person years of blindness over a 10-year period. They concluded that community population screening might be cost effective if targeted at persons aged 40-69-years, and if implemented in urban areas.

For peer review only - http://bmjopen.bmj.com/site/about/guidelines.xhtml

Unlike screening programs for glaucoma, diabetic retinopathy (DR) screening is well established in many high-income countries, and increasingly in LMICs as well. It would seem logical that screening for two major blinding eye diseases, glaucoma and DR, using the same equipment and personnel would be more cost effective. It may be possible to train non-ophthalmic graders who are currently screening for DR to recognize glaucoma using the colour fundus images, reducing the diagnostic burden on scarce, highly trained health care providers in low-resource settings.

The aim of the current study is to develop and test an online course to train nurses, technicians, and non-ophthalmologist physicians working as DR graders in a non-governmental organization (NGO) program in Vietnam to recognize glaucomatous optic nerves from ophthalmic images. The pre- and post-training performance of graders are assessed on a standard test set of optic nerve images drawn from a variety of sources, and compared with the performance of local ophthalmologists not undergoing the same training.

Methods

Approval for the study was obtained from the Ethical Review Board of the Queen's University Belfast in the United Kingdom, and the Ethics Committee of the Hanoi Medical College in Vietnam before the commencement of the study. Participating DR graders and ophthalmologists in Vietnam all provided written informed consent before recruitment into the study.

Patient and Public Involvement statement**:** None

This is an uncontrolled, experimental, before-and-after study in which 43 non-ophthalmic DR graders were trained to screen for glaucoma using optic nerve photos. The online training course was developed specifically for the study and focused on a standardized approach to the pattern recognition of glaucomatous damage to the optic nerve. The study flow was as follows:

For peer review only - http://bmjopen.bmj.com/site/about/guidelines.xhtml

- Non-ophthalmic graders in a non-governmental (NGO) DR program (DR graders) were consented, enrolled and underwent testing on the standard image set at baseline.
- The DR graders then completed the self-paced, online training course. Participants were encouraged to attempt practice questions placed at the end of each module of the course, and those failing to answer these correctly were asked to review the course again.
- DR graders then repeated the test on the standard image set. Images were identical to those at baseline, but were presented in random order. DR grader test scores were compared to baseline and also to those of 29 local ophthalmologists taking part in the NGO DR program, but who did not take the optic nerve grading course.
- Finally, to determine whether further improvement was practical, the non-graders each underwent approximately 20-30 minutes of individually-focused, one-to-one training by a glaucoma specialist (LN), and were tested a third time on the standard image set.

Participants

We enrolled 43 graders currently working in the Orbis and Fred Hollow Foundation (FHF) DR screening project in Vietnam, excluding any ophthalmologists or optometrists. These included nurses, technicians and non-ophthalmic physicians (Table 1). Graders had a previous 3-day training course on grading diabetic retinopathy (DR). After the training, they were encouraged to continue practicing on their own until they were proficient in taking photos and grading these photos for DR. Twenty-nine ophthalmologists in Vietnam from 20 eye hospitals and general hospitals in cities such as Hanoi, Da Nang and Ho Chi Minh City taking part in the project were also recruited for comparison.

For peer review only - http://bmjopen.bmj.com/site/about/guidelines.xhtml

The DR graders had already been trained to grade images for DR through the Orbis and FHF DR projects. Participants were informed that if they chose not to participate in the study, their jobs would not be affected in any way.

Test set of optic nerve images

A total of 60 digital photographic images was used, 33 from eyes with glaucoma and 27 healthy eyes. Among these, 19 images were obtained from the Orbis Vietnam program, 11 from the population-based NICOLA (Northern Ireland Cohort for the Longitudinal Study of Ageing) study and 23 from the Glaucomatous Optic Neuropathy Evaluation (GONE, https://gone-project.com/newgone/) training website. All images were of gradable quality in the opinion of the standard panel of glaucoma specialists, though clarity was not always optimal in order to mimic real world conditions. Images from the GONE website were categorized into difficult, intermediate and easy (Table 1) on the basis of the prior performance of several thousand website users in characterizing them as normal or glaucomatous. [11] The GONE images were graded into normal and abnormal by investigators (NC, AAB, MC, OO, all fellowship-trained glaucoma experts, and TP, a medical retina specialist with a clinical and research focus on image-based screening for eye disease). The images from NICOLA and the Orbis Vietnam program were also sorted into normal and abnormal based on characteristic glaucomatous features of the images by the same group of investigators. When there was internal disagreement between the investigators, the image was replaced with another on which all five agreed.

Although optic nerve images are considered personal data, all images in the test set were fully anonymized and de-identified. Blanket permission for research use of images was obtained through the NICOLA study, Orbis Vietnam DR program and the GONE website, all of which have received prior ethics approval.

For peer review only - http://bmjopen.bmj.com/site/about/guidelines.xhtml

Online training course

The online training course was delivered on the Orbis Cybersight website,(cybersight.org) and consisted of three modules (217 Vietnamese-language PowerPoint slides with voice-over narration in Vietnamese) describing a standardized, step-by-step process of evaluating the healthy and glaucomatous optic nerve head. Module 1 provided a simple introduction to glaucoma. Module 2 introduced the normal optic nerve head and a systematic description of its features, with some reference to images depicting glaucomatous damage. Module 3 described a systematic method for assessing optic nerve head photos to screen for glaucomatous features using "the 5 Cs": Cup to disc ratio, color of the neuro-retinal rim, comparison between the optic nerve heads in a single patient's two eyes, contour of the nerve, and "concerns" (this includes optic disc haemorrhage, and papilledema, or optic nerve oedema with blurred nerve head margins). At the end of modules 2 and 3, trainees were provided 50 normal optic nerve images and 50 images of glaucomatous nerves for practice, all drawn from the same three sources as the test set (the Orbis Vietnam DR program, the NICOLA study and the GONE website).

Optic nerve photos suggestive of other ocular diseases besides glaucoma were excluded from the course, with the exception of potentially fatal conditions affecting the optic nerve, including optic nerve edema as above. All modules were independently assessed and validated by the five investigators listed above (AAB, MC, TP, NC, OO) for content and appropriateness for these cadres of health workers.

Evaluation with the test set

As above, the set consisted of 60 images, which were presented without stereo on the Orbis Cybersight website, and were each graded as "refer" (likely glaucoma) or "do not refer" (normal) by the test-taker. For images graded as "refer," the correct reason had to be selected from a list.

For peer review only - http://bmjopen.bmj.com/site/about/guidelines.xhtml

A score of five marks was given for a correct 'no refer' decision. For a correct 'refer' decision, the participant received a score of 5 marks, and an additional 5 marks for every correct reason given for the referral, ranging from one to six reasons. No marks were deducted for incorrect responses. Each of the 60 questions was worth between 5 and 30 marks (depending on the number of correct reasons for referral), and a total of 470 marks were available on the test. A percentage score was calculated for each grader as the number of marks received divided by 470.

Correct answers were not revealed to the graders. The questions were presented in a random order, which differed between pre-test and post-test, and also from one test-taker to the other. None of the optic nerve images used in the practice test were included in the quiz.

Additional focused one-to-one training

In order to determine if additional, measurable performance improvements could be realized with further, more intensive training, the DR graders each underwent 20-30 minutes of one-to-one training in optic nerve recognition by a glaucoma specialist (LN) to better distinguish presence of glaucomatous optic nerve damage. The training focused on individual areas of deficiency identified by the post-course training and was conducted online due to Covid-related restrictions on face-to-face gatherings in Vietnam at the time. All 43 (100%) DR graders completed the additional training, and all underwent a final round of testing on the standard image set.

Sample size and statistical methods

The minimum sample size of 42 participants (graders) was calculated using the paired sample size formula for qualitative outcome data reported by Hulley et al. [12] with confidence level of 95% and statistical power of 80%. Using the statistics from the GONE website, we estimated that the mean for our study participants at baseline (pre-training) would be at the 5th percentile

For peer review only - http://bmjopen.bmj.com/site/about/guidelines.xhtml

in grading performance, while at the end of the course (post-training), they would be at the 50th percentile. Thus, our study was powered to detect an improvement from a score of 29% to 60% correct answers between baseline and post-training performance on the test set.

True positives (TP) were images correctly graded as glaucomatous optic neuropathy while true negatives (TN) were those correctly graded as 'no glaucoma'. False positive (FP) were images that did not have any features suggestive of glaucomatous optic neuropathy but were incorrectly graded as glaucomatous, while false negatives (FN) were images with glaucomatous features that were incorrectly graded as normal by the test-taker. Sensitivity was then calculated as TP divided by the sum of TP and FN, specificity as TN divided by the sum of TN and FP, positive predictive value (PPV) as TP divided by the sum of TP and FP, and negative predictive value (NPV) as TN divided by the sum of TN and FN. Additionally, area under the receiver operating characteristic (AUC) curve and kappa statistic were computed, with their 95% confidence intervals (95% CI). The performance of DR graders after training was compared to that before training, and also to that of ophthalmologists who had not taken the course.

Since this was a study involving diabetic retinopathy graders and ophthalmologists, we did not involve patients and public in the design or conduct or reporting or dissemination of the study

Results

In total, 43 DR graders participated in the training course and completed both pre and-post training tests, and the additional test after the one-to-one training, while 29 Vietnamese ophthalmologists underwent a single test and did not take part in the training. All DR graders undergoing training completed three rounds of testing.

The mean age (32.6±5.5 years) did not differ from that of the ophthalmologists (32.3±7.3 years, p=0.893, Table 1). There was also no statistically significant difference between the age, sex

For peer review only
For peer review only - http://bmjopen.bmj.com/site/about/guidelines.xhtml

distribution and work experience of DR graders vs ophthalmologists. The DR graders required a mean of 297.8 minutes (144.6), to complete the course according to measurements carried out automatically by the Orbis Cybersight website.

DR graders improved significantly between their mean pre-training percent scores (33.3% ± 14.3%) and their post-training performance score [(55.8% ±12.6%), P <0.001)]. (Table 2) The mean post-training score for DR graders did not differ significantly from that for local ophthalmologists (58.7±15.4%, P=0.384]. These findings, significant improvement between pre and post-training tests and the lack of a significant difference between post-training tests by DR graders and the performance of ophthalmologists, were consistent across optic nerve images from all sources. (Table 2)

With respect to other indices of grading accuracy (Table 3), there was no significant change between DR graders' pre-training [85.5% (83.5 – 87.3)] and post-training sensitivity [80.4% (78.3 – 82.4), p<0.001], while specificity improved significantly [47.8 (44.9 – 50.7) to 79.8 (77.3 – 82.0), p<0.001]. The positive and negative predictive values also improved significantly between pre- and post-training tests, as did the area under the ROC curve [66.6 (64.9 – 68.3) to 80.1 (78.5 – 81.6), p<0.001]. (Table 3). Compared to the 29 ophthalmologists, the 43 DR graders had worse sensitivity, better specificity, worse negative predictive value and worse kappa after training. The positive predictive value and the area under the ROC curve (our pre-specified indicator of overall grading accuracy) did not differ between post-training DR graders and ophthalmologists. (Table 3)

Table 4 shows the predictors of the percentage of correct responses among study participants. In the univariate analysis, age <30 years (p=0.005) and experience < 5 years (p<0.001) predicted higher scores. However, in the multivariate analysis, only experience < 5 years remained significant (p=0.006). The difference between post-training graders and local ophthalmologists remained non-significant when adjusting for other factors.

For peer review only - http://bmjopen.bmj.com/site/about/guidelines.xhtml

The test performance of DR graders (n=43) did not improve significantly after additional focused, one-to-one training, when compared to their performance after the online training. (Table 5) Their mean scores still did not differ from the ophthalmologists after this additional focused training. These patterns were the same for optic nerve images from all sources

Discussion

This uncontrolled, experimental, before-and-after study found a significant improvement in the baseline mean scores of non-ophthalmic DR graders after undergoing a 5-hour training course. The post-course scores of the DR graders were almost double the baseline scores, and did not differ significantly from the scores of general ophthalmologists in Vietnam. This suggests that it is possible to train non-ophthalmic DR graders to screen accurately for glaucoma using optic nerve photos using a short, self-paced online course. Additionally, more intensive one-to-one training by an expert did not result in significant further improvement of grading performance, suggesting encouragingly that results obtained with a convenient and scalable tool like our course approach is what may be readily achievable with such cadres.

In many developing countries, half or more of glaucoma patients present with advanced disease. [13-16] Shortage of providers limits access to eye care and leads to delays in diagnosis and management of glaucoma until late in the disease process, often too late for effective, sight-saving treatment. [17,18] For example, a recent study reported > 60% prevalence of blindness in the operative eye among patients at the time of first glaucoma surgery in rural China.[19] Training non-ophthalmic graders to screen for glaucoma may be an initial step in promoting earlier glaucoma diagnosis in low-resource settings. This has been described as task shifting, a model in which lower cadres of staff are trained to do simpler tasks so that scarcer and more highly-trained workers such as ophthalmologists can concentrate on high-level medical decision making and surgical management. [20-23]

For peer review only - http://bmjopen.bmj.com/site/about/guidelines.xhtml

The graders participating in this study were already trained to detect DR, the most frequent microvascular complication of diabetes and the leading cause of blindness in working-age adults. Studies [24-26] have demonstrated that > 90% of severe vision loss from DR can be prevented through regular screening and prompt referral and treatment. Diabetic screening is more cost-effective compared to no screening or opportunistic screening. [27-28] Glaucoma is the most common cause of irreversible blindness globally [2-29] and glaucoma blindness can similarly be prevented by regular screening of high-risk persons, prompt referral and treatment. Training the same cadres to screen for these two major causes of blindness may further improve the cost-effectiveness and cost-benefit of screening. This model is of particular interest in low-income settings, where traditional eye health services are mainly located in urban centres, often with poor accessibility to rural populations.

The model assessed in the current study differs from that described in previous reports in several ways. Non-expert health workers were trained to recognize glaucoma using an online course, and their performance was objectively measured and compared to their baseline, and to the scores of local ophthalmologists who had not taken the course. Previous studies have used telemedicine to send retinal images to reading centres for expert grading. [30-32] This can be challenging to sustain in low-resource settings, as it still depends on the time and availability of ophthalmologists or other relatively scarce trained experts to review retinal images for evidence of disease. Reading centres can also be logistically complex and expensive to establish and maintain in low resource settings, due to challenges such as the low bandwidth of available internet connections.

An advantage of training non experts as in our current model is that they are more likely to be able to review the images in real time due to lack of competing clinical duties. In delayed grading models, such as those using telemedicine, patients have to be contacted later to notify them of their screening results. Existing studies in LMICs have shown that this can lead to

For peer review only - http://bmjopen.bmj.com/site/about/guidelines.xhtml

substantial loss to follow-up. [33] The RAIDERS study in Rwanda reported that patients receiving immediate feedback on the need for referral demonstrated higher adherence with recommended services compared to participants informed of the results of their screening several days later. [33]

Training with our course significantly improved graders' specificity and positive predictive value. This effect of training is particularly relevant in low-resource settings, where the priority must be to avoid over-burdening already weak health systems with large numbers of false positive referrals. Ausayakhun et al [34] described a somewhat comparable model to ours, of training non-ophthalmologists to deliver point-of-service screening for glaucoma, cataract and age-related macular degeneration, and their reported specificity for glaucoma was approximately 80% using fundus photographs, similar to that observed in the current study.

Although the sensitivity of DR graders decreased slightly after training, their specificity rose substantially. In screening programs for a relatively rare disease such as glaucoma, affecting roughly 2-4% of the population in most studies, avoiding false positives (good specificity) is most important for the cost-effectiveness of the program. For example, assuming a 3% prevalence of glaucoma, the increase in specificity from 47.8% to 79.8% observed after training would reduce the number of false positive referrals from 506 to 196 (61.3% more reduction) in a hypothetical population of 1000 persons, while the observed decline of sensitivity would result in only two more false negative cases." Although this is still a substantial number of false positives, it is fewer than the ophthalmologist would get, as the specificity of the graders post testing exceeds that of the ophthalmologists (79.8 vs 73.8%).

An important determinant of the positive predictive value of a screening program is the burden of disease in the target population. [35] The heavy burden of glaucoma among Asians [36-38] and Africans [39-41] has been well-documented. It is estimated that Asia has the highest number of persons living with glaucoma globally, and that this figure will increase by 79.8%

For peer review only - http://bmjopen.bmj.com/site/about/guidelines.xhtml

(primary open-angle glaucoma) and 58.4% (primary angle closure glaucoma) by 2040. [3] In view of this high glaucoma burden in Asia, and the growing gap between need and supply for ophthalmologists globally, [42] it is clear that new screening models are needed which rely on non-experts. We were able to train diabetic graders to perform at the level of local ophthalmologists, suggesting that our training course can deliver successful and effective task shifting.

An important predictor of correct responses on the test set among study participants was working experience of < 5 years. Lack of a positive effect of cumulative experience may seem counter-intuitive, but we suggest this may reflect better performance among younger and more-recently-trained cadres. Further studies are needed to replicate and better understand this finding.

Although artificial intelligence (AI) has been used successfully for DR screening, [43-44] well trained human graders are crucial in the validation of these AI systems, which must employ locally-derived images and personnel to be most reliable. Moreover, there are substantial existing legal and financial barriers to widespread sustainable use of AI for DR and glaucoma screening in many LMICs. Human systems are needed until these are overcome.

Strengths of this study include an approach to assessing the impact of training which included both a before-after comparison and external validation against the performance of local ophthalmologists. Success of the course against both of these criteria was consistent across images of varying difficulty and drawn from a wide variety of sources. Both the course and the assessment test reflected input from clinical experts, specialists in image-based screening and those engaged professionally in capacity-building for the recognition of glaucomatous optic nerves. A number of widely-used indices of screening accuracy were computed, allowing us to elucidate specific benefits of training in improving specificity.

For peer review only - http://bmjopen.bmj.com/site/about/guidelines.xhtml

This study has limitations as well. The generalizability of our results to other cadres of trainees and other settings is uncertain. Specifically, these results may not be directly applicable to novice graders without prior experience in interpreting fundus images.

Most importantly, further research is needed to better understand the practicality of this training model within the context of a full glaucoma screening program. Additional work is under way to test the model as part of delivering outreach screening for glaucoma in sub-Saharan Africa (SSA), aimed at a broad variety of patients at risk for glaucoma, and not solely those with DR.

In summary, our study shows that it is possible to train non-ophthalmic DR graders quickly and at low cost to screen for glaucoma using optic nerve images. This model may improve glaucoma detection and ultimately reduce the prevalence of blindness from glaucoma in low-resource settings.

For peer review only

For peer review only - http://bmjopen.bmj.com/site/about/guidelines.xhtml

Table 1: Characteristics of DR graders and ophthalmologists participating in a study of glaucomatous optic nerve grading

Characteristics	Ophthalmologists (n=29)	DR graders (n=43)	T-test/Chi-Square (P-value)
**Age (Mean±SD) yrs**	32.6±5.5	32.3±7.3	0.13 (0.893)
<30 years	11 (37.9%)	21 (48.8%)	0.83 (0.361)
≥30 years	18 (62.1%)	22 (51.2%)	
**Sex**			1.99 (0.159)
Female (%)	17 (58.6%)	32 (74.4%)	
Male (%)	12 (41.4%)	11 (25.6%)	
**Job Title**			
Nurse (%)	0 (0.0%)	18 (41.9%)	
Ophthalmologist (%)	29 (100.0%)	0 (0.0%)	
Other physician (%)*	0 (0.0%)	22 (51.2%)	
Technician (%)	0 (0.0%)	3 (6.9%)	
**Work Experience (Mean±SD) yrs**	7.21±5.2	8.22±7.1	0.65 (0.516)
<5 (%)	11 (37.9%)	18 (41.9%)	0.11 (0.739)
≥5 (%)	18 (62.1%)	25 (58.1%)	

*Endocrinologist (5); General Practitioner (14); Radiologist (2); Internist (1)

For peer review only - http://bmjopen.bmj.com/site/about/guidelines.xhtml

Table 2: Accuracy (% Correct vs Gold Standard*) in grading optic nerve images from different sources, comparing DR graders and ophthalmologists

Source of Images	Number of images	% Pre-training Score of graders (A) Mean±SD	% Post-training Score of graders (B) Mean±SD	P-value) (A vs B)	% Scores of Ophthalmologists (C) Mean±SD	P-value (B vs C)
GONE Website						
Easy	8	31.9±17.6	59.2±17.6	<0.001	65.1±21.9	0.216
Moderate	8	25.6±13.3	44.2±19.4	<0.001	48.1±17.8	0.392
Hard	7	22.7±15.9	47.7±22.5	<0.001	46.2±21.8	0.780
Orbis Vietnam DR program	19	47.0±22.7	70.3±12.0	<0.001	70.3±18.2	0.991
NICOLA population-based study in Northern Ireland	11	31.4±20.0	58.7±16.9	<0.001	55.2±22.1	0.443
Other images illustrating specific findings	4	31.0±19.8	51.9±24.1	<0.001	57.5±23.4	0.337
****Total**	60	33.3± 14.3	55.8±12.6	<0.001	58.7±15.4	0.384

*A panel of four fellowship-trained glaucoma specialist ophthalmologists and one medical retina specialist with a clinical and research focus on image-based screening for eye disease (AAB, MC, TP, NC, OO) **The remaining 3 images came from a variety of other sources.

For peer review only - http://bmjopen.bmj.com/site/about/guidelines.xhtml

Table 3: Accuracy in grading optic nerve images, comparing DR graders and ophthalmologists, and calculated against a panel of specialist ophthalmologists (the "gold standard").

Participant Type	Sensitivity (95%CI)	Specificity (95%CI)	Positive Predictive Value (95%CI)	Negative Predictive Value (95%CI)	Kappa (95%CI)	Area under the Receiver-Operator Curve (95%CI)
DR graders, Pre-Training (%)	85.5 (83.5 – 87.3)	47.8 (44.9 – 50.7)	66.7 (65.4 – 68.0)	72.9 (70.1 – 75.6)	34.4 (30.9 – 37.9)	66.6 (64.9 – 68.3)
DR graders, Post-training(%)	80.4 (78.3 – 82.4)	79.8 (77.3 – 82.0)	82.9 (81.2 – 84.5)	76.9 (74.9 – 78.8)	60.0 (56.9 – 63.1)	80.1 (78.5 – 81.6)
P-value Comparing Graders Pre and Post-training	<0.001	<0.001	<0.001	<0.011	<0.001	<0.001
Ophthalmologists (No Training Given) (%)	92.9 (91.1 – 94.4)	73.8 (70.6 – 76.9)	81.3 (79.4 – 83.0)	89.5 (87.1 – 91.5)	67.8 (64.3 – 71.3)	83.4 (81.6 – 85.1)
P-value comparing Post-training graders and Ophthalmologists	<0.001	0.002	>0.05	<0.05	<0.05	>0.05

For peer review only

For peer review only - http://bmjopen.bmj.com/site/about/guidelines.xhtml

Table 4: Predictors of the percentage of correct responses on the test set among study participants

Potential Predictor	Univariate Analysis			Multivariate Analysis		
	Beta	95% Confidence Interval	P-value	Beta	95% Confidence Interval	P-value
Age <30 years	9.0	2.8 – 15.2	0.005	0.4	-8.7 – 9.4	0.938
Male sex	0.4	-6.5 – 7.4	0.899	1.8	-4.7 – 8.3	0.585
Post-training non-ophthalmic grader (Vs ophthalmologist)	-2.9	-9.5 – 3.7	0.384	-3.1	-9.2 – 3.1	0.319
<5 years of work experience	12.5	6.6 – 18.4	<0.001	13.1	3.9 – 22.3	0.006
Nurse (vs "Other physicians," non-ophthalmic grader group only)	-4.5	12.3 – 3.3	0.251			
Pre vs post-training (DR graders only)	0.4	0.1 – 0.6	0.007			

For peer review only

Table 5: Accuracy (% Correct vs Gold Standard*) in grading optic nerve images from different sources: the effect of additional one-one training for DR graders after the online course

Source of Images	Number of images	% Post-training score of graders after online course Mean±SD (A) (n=43)	% Post training score of graders after additional focused one-one training, Mean±SD (B) (n=43)	P-value) (A vs B)	% Scores of Ophthalmologists Mean±SD (C) (n=29)	P-value (B vs C)
GONE Website						
Easy	8	59.2±17.6	60.0±21.6	0.9364	65.1±21.9	0.6463
Moderate	8	44.2±19.4	48.7±21.9	0.6702	48.1±17.8	0.9529
Hard	7	47.7±22.5	49.7±25.8	0.8797	46.2±21.8	0.7886
Orbis Vietnam DR program	19	70.3±12.0	69.8±14.8	0.9096	70.3±18.2	0.9265
NICOLA population-based study in Northern Ireland	11	58.7±16.9	66.7±14.8	0.2514	55.2±22.1	0.1670
Other images illustrating specific findings	4	51.9±24.1	56.1±26.4	0.8220	57.5±23.4	0.9393
****Total**	**60**	**55.8±12.6**	**60.3±16.4**	**0.1573**	**58.7±15.4**	**0.6422**

For peer review only - http://bmjopen.bmj.com/site/about/guidelines.xhtml

Contributorship statement: NC, OO, AB, VC, DC and NP conceived and designed the study. All authors made inputs into the final design of the study. OO drafted the initial course and the test for the graders and ophthalmologists. NC, AB, MK, MC, OO; VC, DC, LN and TP revised the course and test contents and ensured standardization for the study. OO, KF, NC, CR, TH conducted the analysis. OO drafted the initial manuscript. NC, AB, MK, DC, TP, MC, NP and VC made major contributions to the drafting of the manuscript. All authors reviewed it critically for important intellectual content; and approved the final version to be published.

Competing interests: Congdon serves as a paid consultant for Belkin Vision, a company selling devices for the treatment of glaucoma. Kahook has an equity interest in SpyGlass Pharma and receives royalties from New World Medical and Alcon. Olusola Olawoye is a Consortium for Advanced Research and Training in Africa (CARTA) Fellow.

Funding: eXcellence in Ophthalmology and Vision Awards (XOVA Novartis), and Wellcome Trust grant number: 222490/Z/21/Z

Award grant Number: XOVA Novartis: N/A, Wellcome Trust: 222490/Z/21/Z

Data sharing statement: Data are available upon reasonable request

Ethics Approval: Approval for the study was obtained from the Ethical Review Board of the Queen's University Belfast United Kingdom (MHLS 20_98), and the Ethics Committee of the Hanoi Medical College in Vietnam (IRB-VN01.001/IRB 00003121/ FWA 00004148, Approval number 587)

For peer review only

References

1. Flaxman SR, Bourne RRA, Resnikoff S, et al. Global causes of blindness and distance vision impairment 1990-2020: a systematic review and meta-analysis. *Lancet Glob Heal*. 2017;5(12):e1221-e1234. doi:10.1016/S2214-109X(17)30393-5

2. Causes of blindness and vision impairment in 2020 and trends over 30 years, and prevalence of avoidable blindness in relation to VISION 2020: the Right to Sight: an analysis for the Global Burden of Disease Study. *Lancet Glob Heal*. 2021;9(2):e144-e160. doi:10.1016/S2214-109X(20)30489-7

3. Tham YC, Li X, Wong TY, Quigley HA, Aung T, Cheng CY. Global prevalence of glaucoma and projections of glaucoma burden through 2040: A systematic review and meta-analysis. *Ophthalmology*. 2014;121(11). doi:10.1016/j.ophtha.2014.05.013

4. Bourne RRA, Taylor HR, Flaxman SR, et al. Number of people blind or visually impaired by glaucoma worldwide and in world regions 1990 - 2010: A meta-analysis. *PLoS One*. 2016;11(10):1-16. doi:10.1371/journal.pone.0162229

5. Dandona R, Dandona L, Naduvilath TJ, McCarty CA, Rao GN. Utilisation of eyecare services in an urban population in southern India: the Andhra Pradesh eye disease study. *Br J Ophthalmol*. 2000;84(1):22-27. doi:10.1136/bjo.84.1.22

6. Ramakrishnan R, Nirmalan PK, Krishnadas R, et al. Glaucoma in a rural population of Southern India: The Aravind Comprehensive Eye Survey. *Ophthalmology*. 2003;110(8):1484-1490. doi:10.1016/S0161-6420(03)00564-5

7. Burr JM, Mowatt G, Hernández R, et al. The clinical effectiveness and cost-effectiveness of screening for open angle glaucoma: A systematic review and economic evaluation. *Health Technol Assess (Rockv)*. 2007;11(41). doi:10.3310/hta11410

8. Hernandez R, Rabindranath K, Fraser C, Vale L, Blanco AA, Burr JM. Screening for open angle glaucoma: systematic review of cost-effectiveness studies. *J Glaucoma*. 2008;17(3):159-168. doi:10.1097/IJG.0b013e31814b9693

9. Tang J, Liang Y, O'Neill C, Kee F, Jiang J, Congdon N. Cost-effectiveness and cost-utility of population-based glaucoma screening in China: a decision-analytic Markov model. *Lancet Glob Heal*. 2019;7(7):e968-e978. doi:10.1016/S2214-109X(19)30201-3

10. John D, Parikh R. Cost-effectiveness of community screening for glaucoma in rural India: a decision analytical model. *Public Health*. 2018;155:142-151. doi:10.1016/j.puhe.2017.11.004

11. Kong YX, Coote MA, O'Neill EC, Gurria LU, Xie J, Garway-Heath D, Medeiros FA, Crowston JG. Glaucomatous optic neuropathy evaluation project: a standardized internet system for assessing skills in optic disc examination. Clin Exp Ophthalmol. 2011 May-Jun;39(4):308-17. doi: 10.1111/j.1442-9071.2010.02462.x. Epub 2011 Jan 14. PMID: 21070546.

12. Hulley S. *Designing Clinical Research (3rd Ed.) Philadeiphia, PA: Lippioncott Williams, Wilkins*.; 2007.

13. Olawoye O, Kizor-Akaraiwe N, Pons J, et al. Clinical Characteristics and Stage at Presentation of Glaucoma Patients in Sub-Saharan Africa. *J Glaucoma*. June 2022. doi:10.1097/IJG.0000000000002068

14. Gyasi ME, Francis AW, Chen Y, Harrison RSR, Kodjo AR. Presentation of glaucoma in the greater Accra metropolitan area of Ghana. *Ghana Med J*. 2014;48(3):143-147.

For peer review only - http://bmjopen.bmj.com/site/about/guidelines.xhtml

15. Omoti AE, Osahon AI, Waziri-Erameh MJM. Pattern of presentation of primary open-angle glaucoma in Benin City, Nigeria. *Trop Doct*. 2006;36(2):97-100. doi:10.1258/004947506776593323

16. Abdull MM, Gilbert CC, Evans J. Primary open angle glaucoma in northern Nigeria: stage at presentation and acceptance of treatment. *BMC Ophthalmol*. 2015. doi:10.1186/s12886-015-0097-9

17. Mansberger SL, Sheppler C, Barker G, et al. Long-term Comparative Effectiveness of Telemedicine in Providing Diabetic Retinopathy Screening Examinations: A Randomized Clinical Trial. *JAMA Ophthalmol*. 2015;133(5):518-525. doi:10.1001/jamaophthalmol.2015.1

18. Estopinal CB, Ausayakhun S, et al. Access to ophthalmologic care in Thailand: a regional analysis. *Ophthalmic Epidemiol*. 2013;20(5):267-273. doi:10.3109/09286586.2013.821498

19 Yang K, Jin L, Li L*, et al* Preoperative characteristics and compliance with follow-up after trabeculectomy surgery in rural southern China. *British Journal of Ophthalmology* 2017;**101:**131-137.

20. Gichangi M, Kalua K, Barassa E, Eliah E, Lewallen S, Courtright P. Task Shifting for Eye Care in Eastern Africa: General Nurses as Trichiasis Surgeons in Kenya, Malawi, and Tanzania. *Ophthalmic Epidemiol*. 2015;22(3):226-230. doi:10.3109/09286586.2015.1040924

21 Bascaran C, Mwangi N, D'Esposito F, et al. Effectiveness of task-shifting for the detection of diabetic retinopathy in low- and middle-income countries: a rapid review protocol. *Syst Rev*. 2021;10(1):4. doi:10.1186/s13643-020-01553-

22 Shah M, Noor A, Deverell L, Ormsby GM, Harper CA, Keeffe JE. Task sharing in the eye care workforce: Screening, detection, and management of diabetic retinopathy in Pakistan. A case study. *Int J Health Plann Manage*. March 2018. doi:10.1002/hpm.2508

23. Bolme S, Morken TS, Follestad T, Sørensen TL, Austeng D. Task shifting of intraocular injections from physicians to nurses: a randomized single-masked noninferiority study. *Acta Ophthalmol*. 2020;98(2):139-144. doi:10.1111/aos.14184

24. Swanson M. Retinopathy screening in individuals with type 2 diabetes: who, how, how often, and at what cost--an epidemiologic review. *Optometry*. 2005;76(11):636-646. doi:10.1016/j.optm.2005.08.019

25. Cheung N, Mitchell P, Wong TY. Diabetic retinopathy. *Lancet*. 2010;376(9735):124-136. doi:10.1016/S0140-6736(09)62124-3

26. Stewart MW. Treatment of diabetic retinopathy: Recent advances and unresolved challenges. *World J Diabetes*. 2016;7(16):333-341. doi:10.4239/wjd.v7.i16.333

27. Mead A, Burnett S, Davey C. Diabetic retinal screening in the UK. *J R Soc Med*. 2001;94(3):127-129. doi:10.1177/014107680109400307

28. Jones S, Edwards RT. Diabetic retinopathy screening: a systematic review of the economic evidence. *Diabet Med*. 2010;27(3):249-256. doi:10.1111/j.1464-5491.2009.02870.x

29. Quigley HA, Broman AT. The number of people with glaucoma worldwide in 2010 and

For peer review only - http://bmjopen.bmj.com/site/about/guidelines.xhtml

2020. *Br J Ophthalmol*. 2006;90(3):262-267. doi:10.1136/bjo.2005.081224

30. Rathi S, Tsui E, Mehta N, Zahid S, Schuman JS. The Current State of Teleophthalmology in the United States. *Ophthalmology*. 2017;124(12):1729-1734. doi:10.1016/j.ophtha.2017.05.026

31. Hark LA, Katz LJ, Myers JS, et al. Philadelphia Telemedicine Glaucoma Detection and Follow-up Study: Methods and Screening Results. *Am J Ophthalmol*. 2017;181:114-124. doi:10.1016/j.ajo.2017.06.024

32. Joseph S, Kim R, Ravindran RD, Fletcher AE, Ravilla TD. Effectiveness of Teleretinal Imaging-Based Hospital Referral Compared With Universal Referral in Identifying Diabetic Retinopathy: A Cluster Randomized Clinical Trial. *JAMA Ophthalmol*. 2019;137(7):786-792. doi:10.1001/jamaophthalmol.2019.1070

33 Mathenge W, Whitestone N, Nkurikiye J, et al. Impact of Artificial Intelligence Assessment of Diabetic Retinopathy on Referral Service Uptake in a Low-Resource Setting: The RAIDERS Randomized Trial. *Ophthalmol Sci*. 2022;2(4):100168. doi:https://doi.org/10.1016/j.xops.2022.100168

34. Ausayakhun S, Snyder BM, Ausayakhun S, et al. Clinic-Based Eye Disease Screening Using Non-Expert Fundus Photo Graders at the Point of Screening: Diagnostic Validity and Yield. *Am J Ophthalmol*. 2021;227:245-253. doi:10.1016/j.ajo.2021.03.029

35. Chasan JE, Delaune B, Maa AY, Lynch MG. Effect of a teleretinal screening program on eye care use and resources. *JAMA Ophthalmol*. 2014;132(9):1045-1051. doi:10.1001/jamaophthalmol.2014.1051

36. Wang YX, Xu L, Yang H, Jonas JB. Prevalence of glaucoma in North China: the Beijing Eye Study. *Am J Ophthalmol*. 2010;150(6):917-924. doi:10.1016/j.ajo.2010.06.037

37. Casson RJ, Newland HS, Muecke J, et al. Prevalence of glaucoma in rural Myanmar: the Meiktila Eye Study. *Br J Ophthalmol*. 2007;91(6):710-714. doi:10.1136/bjo.2006.107573

38. Song W, Shan L, Cheng F, et al. Prevalence of glaucoma in a rural northern china adult population: a population-based survey in kailu county, inner mongolia. *Ophthalmology*. 2011;118(10):1982-1988. doi:10.1016/j.ophtha.2011.02.050

39. Budenz DL, Barton K, Whiteside-de Vos J, et al. Prevalence of glaucoma in an urban West African population: the Tema Eye Survey. *JAMA Ophthalmol*. 2013;131(5):651-658. doi:10.1001/jamaophthalmol.2013.1686

40. Ashaye A, Ashaolu O, Komolafe O, et al. Prevalence and types of glaucoma among an indigenous African population in southwestern Nigeria. *Investig Ophthalmol & Vis Sci*. 2013;54(12).

41. Rotchford AP, Kirwan JF, Muller MA, Johnson GJ, Roux P. Temba glaucoma study: a population-based cross-sectional survey in urban South Africa. *Ophthalmology*. 2003;110(2):376-382. doi:10.1016/S0161-6420(02)01568-3

42 Resnikoff S, Felch W, Gauthier TM, Spivey B. The number of ophthalmologists in practice and training worldwide: a growing gap despite more than 200,000 practitioners. Br J Ophthalmol. 2012 Jun;96(6):783-7. doi: 10.1136/bjophthalmol-2011-301378. Epub 2012 Mar 26. PMID: 22452836.

43 Bellemo V, Lim ZW, Lim G, Nguyen QD, Xie Y, Yip MYT, Hamzah H, Ho J, Lee XQ, Hsu W, Lee ML, Musonda L, Chandran M, Chipalo-Mutati G, Muma M, Tan GSW, Sivaprasad

For peer review only - http://bmjopen.bmj.com/site/about/guidelines.xhtml

S, Menon G, Wong TY, Ting DSW. Artificial intelligence using deep learning to screen for referable and vision-threatening diabetic retinopathy in Africa: a clinical validation study. Lancet Digit Health. 2019 May;1(1):e35-e44. doi: 10.1016/S2589-7500(19)30004-4. Epub 2019 May 2. PMID: 33323239.

44 Hansen MB, Abràmoff MD, Folk JC, Mathenge W, Bastawrous A, Peto T (2015) Results of Automated Retinal Image Analysis for Detection of Diabetic Retinopathy from the Nakuru Study, Kenya. PLoS ONE 10(10): e0139148. https://doi.org/10.1371/journal.pone.0139148

For peer review only

For peer review only - http://bmjopen.bmj.com/site/about/guidelines.xhtml

STARD Checklist

Section & Topic	No	Item	Reported on page #
**TITLE OR ABSTRACT**			
	**1**	Identification as a study of diagnostic accuracy using at least one measure of accuracy (such as sensitivity, specificity, predictive values, or AUC)	Page 2
**ABSTRACT**			
	**2**	Structured summary of study design, methods, results, and conclusions (for specific guidance, see STARD for Abstracts)	Page 2
**INTRODUCTION**			
	**3**	Scientific and clinical background, including the intended use and clinical role of the index test	Page 5 and 6
	**4**	Study objectives and hypotheses	Page 6
**METHODS**			
Study design	**5**	Whether data collection was planned before the index test and reference standard were performed (prospective study) or after (retrospective study)	Page 6 and 7
Participants	**6**	Eligibility criteria	Page 7
	**7**	On what basis potentially eligible participants were identified (such as symptoms, results from previous tests, inclusion in registry)	Page 7
	**8**	Where and when potentially eligible participants were identified (setting, location and dates)	Page 7
	**9**	Whether participants formed a consecutive, random or convenience series	Page 7
Test methods	**10a**	Index test, in sufficient detail to allow replication	Pages 8-10
	**10b**	Reference standard, in sufficient detail to allow replication	Pages 9-10
	**11**	Rationale for choosing the reference standard (if alternatives exist)	
	**12a**	Definition of and rationale for test positivity cut-offs or result categories of the index test, distinguishing pre-specified from exploratory	Page 10-11
	**12b**	Definition of and rationale for test positivity cut-offs or result categories of the reference standard, distinguishing pre-specified from exploratory	
	**13a**	Whether clinical information and reference standard results were available to the performers/readers of the index test	NA
	**13b**	Whether clinical information and index test results were available to the assessors of the reference standard	9-11
Analysis	**14**	Methods for estimating or comparing measures of diagnostic accuracy	10-11
	**15**	How indeterminate index test or reference standard results were handled	NA
	**16**	How missing data on the index test and reference standard were handled	NA
	**17**	Any analyses of variability in diagnostic accuracy, distinguishing pre-specified from exploratory	
	**18**	Intended sample size and how it was determined	10-11
**RESULTS**			
Participants	**19**	Flow of participants, using a diagram	No diagram but well described on page 7
	**20**	Baseline demographic and clinical characteristics of participants	Page 11, table 1
	**21a**	Distribution of severity of disease in those with the target condition	NA
	**21b**	Distribution of alternative diagnoses in those without the target condition	NA
	**22**	Time interval and any clinical interventions between index test and reference standard	Table 4
Test results	**23**	Cross tabulation of the index test results (or their distribution) by the results of the reference standard	Table 2
	**24**	Estimates of diagnostic accuracy and their precision (such as 95% confidence intervals)	Table 3
	**25**	Any adverse events from performing the index test or the reference standard	NA
**DISCUSSION**			
	**26**	Study limitations, including sources of potential bias, statistical uncertainty, and generalisability	Page 17

For peer review only - http://bmjopen.bmj.com/site/about/guidelines.xhtml

For peer review only

	**27**	Implications for practice, including the intended use and clinical role of the index test	Pages 16 -17
**OTHER INFORMATION**			
	**28**	Registration number and name of registry	NCT05151757 ClinicalTrials.gov
	**29**	Where the full study protocol can be accessed	
	**30**	Sources of funding and other support; role of funders	eXcellence in Ophthalmology and Vision Awards (XOVA Novartis)

For peer review only

For peer review only
